# Discrimination of Pancreatic Serous Cystadenomas From Mucinous Cystadenomas With CT Textural Features: Based on Machine Learning

**DOI:** 10.3389/fonc.2019.00494

**Published:** 2019-06-12

**Authors:** Jing Yang, Xinli Guo, Xuejin Ou, Weiwei Zhang, Xuelei Ma

**Affiliations:** ^1^State Key Laboratory of Biotherapy, Department of Biotherapy, West China Hospital, Cancer Center, Sichuan University, Chengdu, China; ^2^West China Hospital, West China School of Medicine, Sichuan University, Chengdu, China; ^3^Department of Radiology, West China Hospital, Sichuan University, Chengdu, China

**Keywords:** pancreas, cystadenoma, diagnosis, multidetector computed tomography, machine learning

## Abstract

**Objectives:** This study was designed to estimate the performance of textural features derived from contrast-enhanced CT in the differential diagnosis of pancreatic serous cystadenomas and pancreatic mucinous cystadenomas.

**Methods:** Fifty-three patients with pancreatic serous cystadenoma and 25 patients with pancreatic mucinous cystadenoma were included. Textural parameters of the pancreatic neoplasms were extracted using the LIFEx software, and were analyzed using random forest and Least Absolute Shrinkage and Selection Operator (LASSO) methods. Patients were randomly divided into training and validation sets with a ratio of 4:1; random forest method was adopted to constructed a diagnostic prediction model. Scoring metrics included sensitivity, specificity, accuracy, and AUC.

**Results:** Radiomics features extracted from contrast-enhanced CT were able to discriminate pancreatic mucinous cystadenomas from serous cystadenomas in both the training group (slice thickness of 2 mm, AUC 0.77, sensitivity 0.95, specificity 0.83, accuracy 0.85; slice thickness of 5 mm, AUC 0.72, sensitivity 0.90, specificity 0.84, accuracy 0.86) and the validation group (slice thickness of 2 mm, AUC 0.66, sensitivity 0.86, specificity 0.71, accuracy 0.74; slice thickness of 5 mm, AUC 0.75, sensitivity 0.85, specificity 0.83, accuracy 0.83).

**Conclusions:** In conclusion, our study provided preliminary evidence that textural features derived from CT images were useful in differential diagnosis of pancreatic mucinous cystadenomas and serous cystadenomas, which may provide a non-invasive approach to determine whether surgery is needed in clinical practice. However, multicentre studies with larger sample size are needed to confirm these results.

## Introduction

Pancreatic cysts can be classified into non-neoplastic and neoplastic subtypes (1). Serous cystadenomas, mucinous cystadenomas, and intraductal papillary mucinous neoplasms constitute a majority of the neoplastic subtype encountered in practice (2, 3). Serous cystadenomas are glycogen-rich lesions arising from cuboidal epithelium, which are considered benign and are most found incidentally (4, 5). A multinational study including 2,622 patients diagnosed with serous cystadenoma between 1990 and 2014 has reported that serous cystadenomas occur more frequently in women (74%) at a median age of 58 years (16–99 years) (6). Given the benign nature of serous cystadenomas, surgical intervention is conserved unless symptomatic or the diagnosis remains doubtful. The diagnosis of mucinous cystadenomas rests on the presence of ovarian-type stroma. About 76% of the patients with mucinous cystadenoma are symptomatic at the time of diagnosis, and the most common symptom is non-specific abdominal pain (7). In contrast to serous cystadenomas, mucinous cystadenomas have considerable malignant potential, and therefore guidelines recommend resection for all surgically fit patients. In consideration of the different treatment principles of the two diseases, preoperative differential diagnosis is crucial.

Cross-sectional imaging techniques play an important role in the differential diagnosis of pancreatic cystic neoplasms (8, 9). Computed tomography (CT) is indicated in all patients with pancreatic cystic lesion, and is the most common imaging modality used for differential diagnosis and for identifying signs suggestive of malignancy. Previous studies have indicated that CT appearance including lesion size, location, calcifications, wall enhancement, lobulated contour, thickness of the wall, and presence of septation may helpful in the differential diagnosis between pancreatic serous and mucinous cystadenomas (5, 10, 11). However, the accuracy for the specific diagnosis based on morphological characteristics in CT images is limited even when reviewer certainty is high (12). Recently, texture analysis has been studied in several types of tumor, and it has been proposed that textual parameters of CT images can provide both diagnostic and prognostic information (13, 14). Furthermore, a retrospective study has reported that two-dimensional texture analysis is a potential method to differentiate pancreatic lymphoma and pancreatic adenocarcinoma (15). In this study, we firstly evaluated the performance of three-dimensional texture analysis based on contrast-enhanced CT images in the differential diagnosis of pancreatic mucinous cystadenomas and serous cystadenomas.

## Materials and Methods

### Subjects

**Figure 1:**
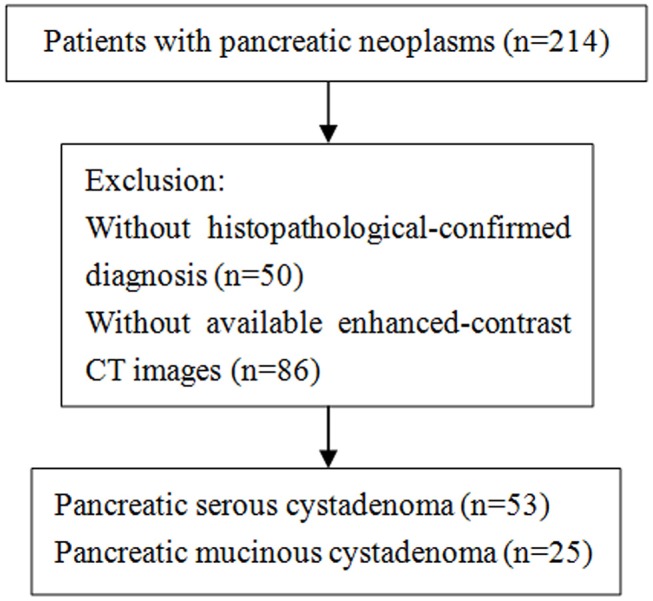
Flowchart shows selection of study population and exclusion criteria.

This study was approved by the Ethics Administration Office of West China Hospital, Sichuan University and no written informed consent was required. We retrospectively reviewed patients who were diagnosed with pancreatic mucinous or serous cystadenoma at our hospital between January 2013 and May 2018, and 214 patients were identified. Patients were considered eligible based on the following criteria: (1) histological-confirmed by means of surgical procedure; (2) contrast-enhanced CT scans (slice thickness of 2 mm or 5 mm) were performed before biopsy and surgical intervention. Of the 214 patients, 50 patients without pathological-confirmed diagnosis were excluded. Subsequently, an additional group of 86 patients was excluded because they had no available enhanced-contrast CT images. Finally, our study population is consisted of 25 patients with mucinous cystadenoma and 53 patients with serous cystadenoma. The selection process was shown in the Figure 1.

### CT Examinations

Contrast-enhanced abdominal CT examinations were performed before the beginning of any treatment, using 64-MDCT scanner (Brilliance64, Philips Medical Systems, Eindhoven, The Netherlands) or 128-MDCT scanner (Somatom Definition AS+, Siemens Healthcare Sector, Forchheim, Germany). All CT examinations were performed with the following parameters: 120 kVp; 200–250 mAs; pitch 0.75–1.0; rotation time 0.5–0.75 s; collimation 0.625 mm; section thickness 2.0 or 5.0 mm. Nonionic contrast material (1.5–2.0 mL/kg) was injected intravenously at a rate of 3 mL/s. Images were obtained during the arterial phase and the portal vein phase, at the times of trigger (trigger threshold of the aorta reaching 100 HU) and 30 s after the trigger, respectively.

### Computerized Texture Analysis

**Figure 2:**
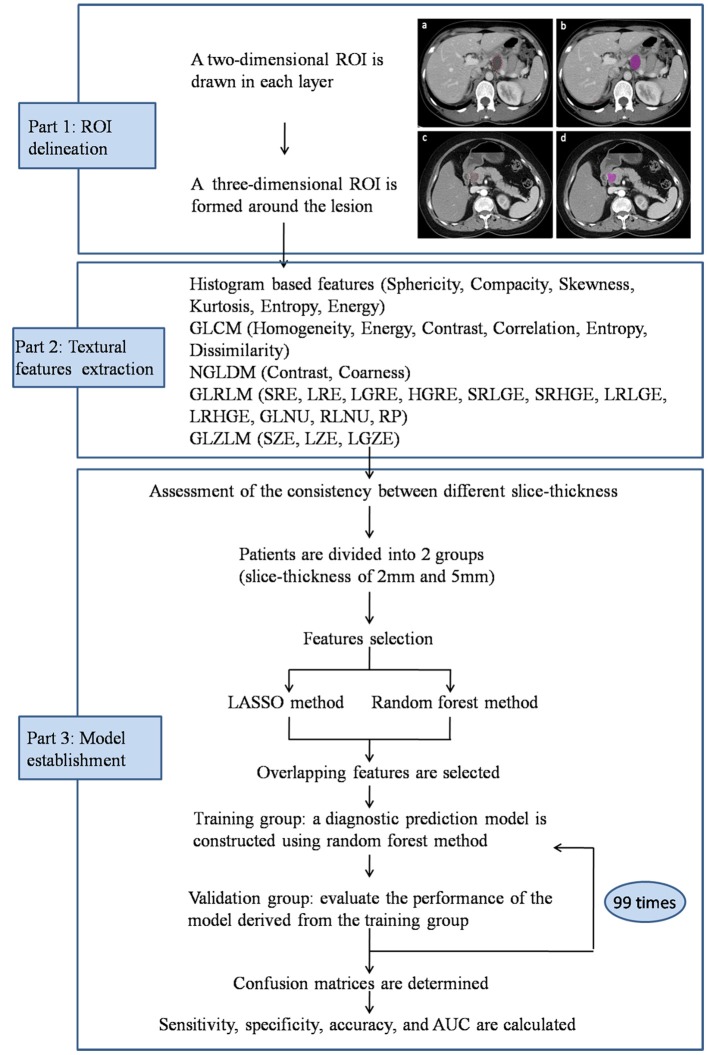
Workflow of image processing and machine learning. Portal vein phase CT images of a patient with histopathologically proved pancreatic mucinous cystadenoma **(a,b)** and pancreatic serous cystadenoma **(c,d)**.

The textural parameters were obtained using Local Image features Extraction (LIFEx) software in the portal vein phase CT images (16). A two-dimensional region of interest (ROI) was delineated around the boundary of tumor lesion in each layer of transaxial CT images to form a three-dimensional ROI. ROIs were drawn independently by two radiologists, who were unaware of the diagnosis of patients. Minimum, maximum, mean, and standard deviation of the density values inside the ROI were calculated. From these primary calculations, geometry based and histogram based features, the Gray-level co-occurrence matrix (GLCM), the Neighborhood gray-level different matrix (NGLDM), the Gray level run length matrix (GLRLM) and the Gray level zone length matrix (GLZLM) were obtained (Figure 2).

### Statistical Analysis

Inter-observer agreement and inter-slice thickness agreement was evaluated between two readers using paired samples *t*-test. The textural parameters derived from the ROIs were analyzed to assess the inter-observer agreement of the ROIs between observers A and B. Of the 78 patients, 42 had both CT images with slice thickness of 2 mm and 5 mm, therefore, we assessed the consistency of the textural parameters between different slice thicknesses using the data from those 42 patients (Supplementary Table 1). Considering the poor consistency between different slice thicknesses, patients with both slice thicknesses of 2 and 5 mm were studied separately. Of the 78 patients, 18 with mucinous cystadenomas and 36 with serous cystadenomas had CT images of 2 mm slice thicknesses; 17 patients with mucinous cystadenomas and 49 patients with serous cystadenomas had CT images of 5 mm slice thicknesses.

The parameters derived from texture analysis and several clinicopathological characteristic (age, gender, size, location of lesions, enhancement of peripheral wall, mural nodules, and calcification of lesions) were analyzed using random forest and Least Absolute Shrinkage and Selection Operator (LASSO) methods (17). In the group of 2 mm slice thickness, 22 parameters were obtained using the random forest analysis and 12 parameters were obtained using LASSO method; 5 overlapping parameters were discovered. In the group of 5 mm slice thickness, 18 parameters were obtained using the random forest analysis and 14 parameters were obtained using LASSO method; 4 overlapping parameters were discovered. Those selected textural parameters were given as mean ± standard deviation. Statistical differences of textural parameters were analyzed using independent sample *t*-test. A *p*-value of <0.05 was considered significant.

Subsequently, we constructed a diagnostic prediction model with the overlapping parameters using random forest method (18). Random forest method involves a combination of multiple classification and regression trees that are independent diagnostic algorithms. Patients were randomly divided into training and validation sets with a ratio of 4:1. In the training group, as initial step, an exploratory data analysis of the textural parameters was performed across the two types of diseases. Then, the validation set was employed to evaluate the performance of the model derived from the training group. The procedure was repeated for 100 cycles with different case assignments to appraise the robustness of the methods. A confusion matrix was determined, and sensitivity, specificity, accuracy, and the areas under the receiver operating characteristic curve (AUC) were calculated from the confusion matrix to evaluate the discriminative ability of the model. Statistical analyses were performed using the PYTHON software (Sklearn package).

## Results

### Patient Characteristics

Fifteen-three patients with serous cystadenoma and 25 patients with mucinous cystadenoma were included in this study. The median age was 52 (range from 29 to 73) years in patients with serous cystadenoma, and was 45 years (range from 28 to 68) in patients with mucinous cystadenoma. There were 14 (26.4%) males and 39 (73.6%) females in the serous group and 7 (28.0%) males and 18 (72.0%) females in the mucinous group. The lesions of half of the patients with mucinous cystadenoma were located in the head of pancreas and the lesions of other patients were located in the body or the tail of pancreas. In 4 (16.0%) patients with serous cystadenoma, the lesions were located within the pancreatic head, whereas in 21 (84.0%) patients the lesions were in the body or the tail of pancreas. The mean size of serous cystadenomas were 3.48 cm (range 1.00–8.00 cm), and the mean size of mucinous cystadenomas were 5.93 cm (range 2.00–12.00 cm). The characteristics of the patients were summarized in the Table 1.

**Table 1:** Characteristics of the patients.

**Characteristics**	**Serous cystic neoplasms**	**Mucinous cystic neoplasms**
**AGE (YEARS)**		
Median (range)	52 (29–73)	45 (28–68)
**GENDER**		
Male	14 (26.4%)	7 (28.0%)
Female	39 (73.6%)	18 (72.0%)
**LOCATION**		
Head	26 (49.1%)	4 (16.0%)
Body or tail	27 (50.9%)	21 (84.0%)
Mean size (range) (cm)	3.48 (1.00–8.00)	5.93 (2.00–12.00)

### Parameters Selection and Random Forest Model

**Figure 3:**
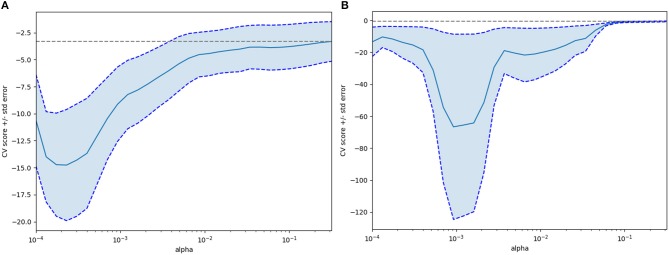
The Lasso (least absolute shrinkage and selection operator) process for selecting features after extraction of textural features (**A**, 2 mm slice thickness group; **B**, 5 mm slice thickness group).

Textural features and baseline characteristics were analyzed using LASSO and random forest method. Five overlapping parameters were discovered in the group of 2 mm slice thickness, including GLRLM_Short-nun high gray-level emphasis (SRHGE), GLRLM_Gray-level non-uniformity (GLNU), GLRLM_Run length non-uniformity (RLNU), GLZLM_Long-zone emphasis (LZE) and GLZLM_Short-zone high gray-level emphasis (SZHGE), while in the group of 5 mm slice thickness, 4 overlapping parameters, including GLRLM_GLNU, GLRLM_RLNU, GLZLM_ Long-zone high gray-level emphasis (LZHGE) and GLZLM_ Zone length non-uniformity (ZLNU) were discovered. The LASSO process was shown in the Figure 3. Table 2 summarize the comparison of those textural features between pancreatic serous cystadenomas and mucinous cystadenomas. A good consistency between ROIs delineated by observers A and B was shown in the Supplementary Table 2.

**Table 2:** Comparison of pancreatic serous cystadenoma and mucinous cystadenoma using overlapping textural parameters selected by lasso method and random forest method.

**Slice thickness**	**Parameters**	**Serous cystadenoma** **(Mean ± standard deviation)**	**Mucinous cystadenoma** **(Mean ± standard deviation)**	***p*-value**
2 mm	GLRLM_ SRHGE	9618.45 ± 110.29	8994.58 ± 164.91	**0.002**
	GLRLM_ GLNU	1708.81 ± 350.35	19224.34 ± 6939.86	**0.022**
	GLRLM_RLNU	9022.23 ± 1774.52	64048.26 ± 23074.55	**0.029**
	GLZLM_LZE	20934.36 ± 5788.32	62766.20 ± 20660.20	0.066
	GLZLM_SZHGE	6492.92 ± 55.31	5880.79 ± 367.91	0.117
5 mm	GLRLM_ GLNU	982.43 ± 201.15	10572.27 ± 3414.19	**0.013**
	GLRLM_RLNU	4468.52 ± 982.31	32363.30 ± 10314.21	**0.016**
	GLZLM_LZHGE	175851321.56 ± 49625896.38	1353775484.89 ± 482202975.93	**0.027**
	GLZLM_ZLNU	208.25 ± 40.94	1120.54 ± 341.00	**0.017**

*GLRLM, Gray level run length matrix; GLZLM, Gray level zone length matrix; SRHGE, Short-nun high gray-level emphasis; GLNU, Gray-level non-uniformity; RLNU, Run length non-uniformity; LZE, Long-zone emphasis; SZHGE, Short-zone high gray-level emphasis; LZHGE, Long-zone high gray-level emphasis; ZLNU, Zone length non-uniformity. The bold values indicate that the corresponding textural features of the two groups are significantly different*.

Subsequently, differential diagnosis ability of these parameters was analyzed using the random forest model. In both groups of patients with CT images of 2 mm and 5 mm slice thickness, the results revealed that textural features were able to discriminate between the mucinous cystadenoma and the serous cystadenoma. In the group of 2 mm slice thickness, the sensitivity was 0.95, the specificity was 0.83, the accuracy was 0.85, and the AUC was 0.77 for the training set; the sensitivity was 0.86, the specificity was 0.71, the accuracy was 0.74, and the AUC was 0.66 for the validation set (Table 2). In the training set of 5 mm slice thickness group, the sensitivity, specificity, accuracy and AUC of the training group were 0.90, 0.84, 0.86, and 0.72, respectively, while in the validation set, the sensitivity, specificity, accuracy and AUC were 0.85, 0.83, 0.83, and 0.75, respectively (Table 3).

**Table 3:** The results derived from random forest model.

**Slice thickness**	**Group**	**Confusion matrix**		**Sensitivity**	**Specificity**	**Accuracy**	**AUC**
2 mm	Training	71	56	0.95	0.83	0.85	0.77
		4	269				
	Validation	19	34	0.86	0.71	0.74	0.66
		3	84				
5 mm	Training	57	65	0.90	0.84	0.86	0.72
		6	362				
	Validation	25	23	0.85	0.83	0.83	0.75
		6	116				

*AUC, area under the curve*.

## Discussion

Given the benign nature of pancreatic serous cystadenomas and malignant potential of mucinous cystadenomas, resection is not suggested for most of the patients with serous cystadenoma while surgical treatment is recommended for all surgical fit patients with mucinous cystadenoma (19). Therefore, preoperative differential diagnosis is critical (19, 20). Currently, cross-sectional imaging, endoscopic ultrasound (EUS), fine-needle aspiration (FNA) biopsy and cyst fluid analysis were frequently employed to assist in the differential diagnosis (1). EUS with cyst fluid analysis is the most important mean to distinguish pancreatic mucinous cystadenomas from serous cystadenomas (1). A cyst fluid carcinoembryonic antigen (CEA) level > 192 ng/mL has been reported to be useful for identification of mucinous cystadenomas, with a sensitivity of 73% and specificity of 84% (21). However, cyst fluid analysis is limited by its invasiveness. Cross-sectional imaging offers the possibility of characterizing lesions in a non-invasive way. In this study, textural features derived from CT images were able to differentiate between pancreatic mucinous cystadenomas and serous cystadenomas, with AUC of 0.73 and 0.70 in the training group and the validation group, respectively.

Previous studies have already assessed the value of CT images in differential diagnosis for pancreatic serous cystadenomas and mucinous cystadenomas. Most of them analyze the qualitative features, and the performance of different readers using morphological characteristics of CT images to evaluate pancreatic cystic lesions is controversial. A previous study has reported that reviews are able to correctly classify 93% of the serous cystadenoma cases and 95% of the mucinous cystadenoma cases (22). Nevertheless, another study has suggested that CT appearance is an insensitive tool for differentiating mucinous and serous tumors and reviewers correctly diagnosis serous neoplasms in only 23–41% of cases (23). More recently, a retrospective study have concluded that location in the head of pancreas, lobulated contour and without wall enhancement are specific characteristics for macrocystic serous cystadenomas. In this study, the specificity of 100% was achieved when three of these characteristics were combined, however, the sensitivity was only 33–58% (10). The interpretation of morphological characteristics for medical images much depends on education, expertise and experiences of observers, so the results of different studies vary a lot.

As a key component of radiomics, texture analysis could provide numerous quantitative and semi-quantitative parameters and reflect the heterogeneity of tissues, which is known to have important implications in cancer research (16). Correlations between textural features and pathological phenotype of lesions have been investigated in previous studies. In a cohort of 534 patients with lung lesion, FDG PET and CT textural features were proposed to be able to differentiate primary and metastatic lesions of lung (24). Mammographic texture analysis was suggested to be a reliable technique for differential diagnosis of benign and malignant breast tumors (25). Furthermore, in a study of pancreas lesions, two-dimensional texture analysis based on CT images was shown to be a feasible quantitative technique for the differential diagnosis of lymphoma and adenocarcinoma (15). Similarly, in this study, we demonstrated similar results that textural features derived from CT images were potential biomarkers to distinguish mucinous cystadenomas from serous cystadenomas. In addition, we randomly divided the patients into training and validation groups and displayed the differential diagnostic performance of textural features in validation group, which suggested that texture analysis may have a good application prospect in clinical practice. The robustness of the procedure was tested by repeating for 100 times with different assignments, which can avoid selection bias in the analysis process.

A previous study investigated the differential diagnosis ability of contrast-enhanced CT images in the differential diagnosis of pancreatic neoplasms has suggested that both arterial phase and portal venous phase CT images are useful (15). Contrast-enhanced CT images of portal venous phase were used in our study. Certainty, the enhancement phases may affect the textural parameters of tumor lesions, but their impact on the differential diagnosis still needs to be further validated. In this study, we also evaluated the consistency of textural features of CT with different slice thickness and found that there was a good correlation between textural parameters obtained from CT images of 2 mm and 5 mm, but the consistency was poor. Several previous studies neglected the heterogeneity of CT images with different slice thickness and mixed the CT with different slice thickness together for analysis (26). The value of contrast-enhanced CT with a slice thickness of 2 mm or 5 mm in differential diagnosis shows little difference, but we suggest that Ct images with different slice thicknesses cannot be mixed. However, more studies are needed to confirm the results of our study.

In general, the heterogeneity of tissue is composed of multiple texture patterns, so a single textural parameter cannot fully display the gross textural characteristics of tumor (27). In the preliminary analysis of this study, we also tried to analyze individual factors, and the results were not satisfactory. In consideration of this, a complex of integrated different textural parameters is required to represent gross texture of tumor more comprehensively. Random forest model, a powerful machine-learning approach, has proved successful in classifying subjects into the correct group (28, 29). Previous studies have also indicated that random forest model could be used in the analysis of textural features (29, 30). In this study, random forest model was able to discriminate between pancreatic mucinous cystadenomas and serous cystadenomas.

Our study has several limitations. Firstly, this is a retrospective study with a small sample size. The robustness of the results was tested by repeating for 100 times with different assignments. Secondly, in order to ensure the accuracy of the diagnosis, all the patients in our department have been operated. However, this is unavoidable due to ethical issue. Thirdly, there is subjectivity in the process of manually outlining the tumor boundary. Therefore, prospective studies with a large population are expected to confirm the present findings.

In conclusion, our study provided preliminary evidence that analysis texture of lesions in CT images was a reliable method to differentiate diagnosis of pancreatic mucinous cystadenomas and serous cystadenomas, which may provide a convenient, non-invasive and repeatable approach to determine whether surgery is needed in clinical practice. However, multicentre studies with larger sample size are needed to confirm these results.

## Data Availability

The datasets for this manuscript are not publicly available. The data used to support the findings of this study are available from the corresponding author upon request.

## Ethics Statement

This study was approved by the Ethics Administration Office of West China Hospital, Sichuan University and no written informed consent was required.

## Author Contributions

JY designed the study, performed the data analysis, and drafted the manuscript. XG and XO performed the data analysis and drafted the manuscript. WZ extracted the data. XM designed the study. All authors read and approved the final manuscript.

### Conflict of Interest Statement

The authors declare that the research was conducted in the absence of any commercial or financial relationships that could be construed as a potential conflict of interest.
