# Supplementary material for: Discrimination of Pancreatic Serous Cystadenomas From Mucinous Cystadenomas With CT Textural Features: Based on Machine Learning
Source: Front Oncol. 2019 Jun 12;9:494. doi: 10.3389/fonc.2019.00494 (PMC6581751; doi:10.3389/fonc.2019.00494)
Supplement: Supplementary file 1 [file Table_1.DOCX]

**Supplementary Table 1: Caparison of the parameters derived from CT images with different slice-thickness**

| **Parameters** | **Slice-thickness of 2mm**  **(Mean±standard deviation)** | **Slice-thickness of 5mm**  **(Mean±standard deviation)** | **p value**  **(Paired samples t-test)** | **Pearson correlation coefficient** |
| --- | --- | --- | --- | --- |
| **HISTO_ Skewness** | 0.58±0.54 | 0.67±0.64 | 0.207 | 0.716 |
| **HISTO_Kurtosis** | 5.17±3.00 | 5.11±3.02 | 0.852 | 0.761 |
| **HISTO_Energy** | 0.15±0.036 | 0.18±0.062 | **<0.001** | 0.843 |
| **SHAPE_VolumemL** | 45.77±124.74 | 44.13±111.91 | 0.452 | 0.996 |
| **SHAPE_Volumevx** | 52012.88±129444.36 | 20508.19±48081.45 | **0.017** | 0.995 |
| **SHAPE_Compacity** | 4.23±3.10 | 1.85±1.46 | **<0.001** | 0.976 |
| **GLCM_Homogeneity** | 0.50±0.048 | 0.54±0.08 | **<0.001** | 0.729 |
| **GLCM_Energy** | 0.028±0.013 | 0.048±0.029 | **<0.001** | 0.925 |
| **GLRLM_SRE** | 0.87±0.037 | 0.85±0.059 | **<0.001** | 0.913 |
| **GLRLM_LRE** | 1.78±0.32 | 2.08±0.67 | **<0.001** | 0.941 |
| **GLRLM_HGRE** | 10934.85±576.74 | 10897.40±844.32 | 0.706 | 0.654 |
| **GLRLM_SRHGE** | 9511.75±715.19 | 9276.19±1054.97 | **0.013** | 0.849 |
| **GLRLM_LRHGE** | 19356.28±3329.18 | 22517.58±7076.70 | **<0.001** | 0.895 |
| **GLRLM_GLNU** | 6932.17±18534.15 | 3213.22±8241.69 | **0.025** | 0.997 |
| **GLRLM_RLNU** | 25673.35±60578.66 | 8175.02±16720.01 | **0.014** | 0.992 |
| **GLRLM_RP** | 0.83±0.048 | 0.81±0.077 | **<0.001** | 0.913 |
| **NGLDM_Coarseness** | 0.0026±0.0047 | 0.0053±0.0081 | **<0.001** | 0.918 |
| **GLZLM_LZE** | 36794.52±64658.17 | 51170.53±133902.97 | 0.745 | 0.579 |
| **GLZLM_HGZE** | 11128.88±560.70 | 11060.47±734.58 | 0.402 | 0.736 |
| **GLZLM_LZHGE** | 390060330.97±675554724.07 | 534390179.61±1382331895.8 | 0.414 | 0.581 |
| **GLZLM_GLNU** | 397.23±980.61 | 149.49±343.17 | **0.016** | 0.995 |
| **GLZLM_ZLNU** | 1044.03±2154.28 | 281.18±474.18 | **0.006** | 0.972 |

Abbreviations: GLRLM, Gray level run length matrix; HGRE, High gray-level run emphasis; SRHGE, Short-nun high gray-level emphasis; LRHGE, Long-run high gray-level emphasis; GLNU, Gray-level non-uniformity; RLNU, Run length non-uniformity; RP, Run Percentage; GLZLM, Gray level zone length matrix; LZE, Long-zone emphasis; HGZE, High gray-level zone emphasis; LZHGE, Long-zone high gray-level emphasis; ZLNU, Zone length non-uniformity.

**Supplementary Table 2: Textural parameters of the ROIs delineated by readers A and B.**

| **Parameters** | **Reader A**  **(Mean±standard deviation)** | **Reader B**  **(Mean±standard deviation)** | **p value**  **(Paired samples t-test)** | **Pearson correlation coefficient** |
| --- | --- | --- | --- | --- |
| **GLRLM_ SRHGE** | 9489.97±761.08 | 9472.22±739.09 | 0.656 | 0.896 |
| **GLRLM_ GLNU** | 4666.03±11237.45 | 5545.98±14830.81 | 0.235 | 0.917 |
| **GLRLM_RLNU** | 17544.47±35052.28 | 21510.45±49326.61 | 0.250 | 0.805 |
| **GLZLM_LZE** | 34432.83±80029.31 | 39358.32±83857.86 | 0.101 | 0.952 |
| **GLZLM_SZHGE** | 6371.60±635.74 | 6331.24±631.19 | 0.302 | 0.859 |
| **GLZLM_LZHGE** | 364202996.25±830285898.26 | 414166483.06±882548236.47 | 0.119 | 0.951 |
| **GLZLM_ZLNU** | 724.02±1228.25 | 898.55±1758.00 | 0.199 | 0.750 |

Abbreviations: GLRLM, Gray level run length matrix; SRHGE, Short-nun high gray-level emphasis; GLNU, Gray-level non-uniformity; RLNU, Run length non-uniformity; GLZLM, Gray level zone length matrix; LZE, Long-zone emphasis; SZHGE, Short-zone high gray-level emphasis; LZHGE, Long-zone high gray-level emphasis; ZLNU, Zone length non-uniformity.
